# Measuring magnetic hysteresis curves with polarized soft X-ray resonant reflectivity

**DOI:** 10.1107/S160057752400119X

**Published:** 2024-04-10

**Authors:** Raymond Fan, Kiranjot, Razan O. M. Aboljadayel, Kalel Alsaeed, Peter Bencok, David M. Burn, Aidan T. Hindmarch, Paul Steadman

**Affiliations:** a Diamond Light Source, Harwell Science and Innovation Campus, Didcot, Oxon OX11 0DE, United Kingdom; b Department of Physics, Durham University, Durham DH1 3LE, United Kingdom; Bhabha Atomic Research Centre, India

**Keywords:** reflectivity, scattering, magnetism, soft X-rays, polarization, hysteresis, helicity, thin films

## Abstract

Calculations and measurements of polarization-dependent soft X-ray scattering intensity are presented during a magnetic hysteresis cycle. It is confirmed that the dependence of the intensity on the magnetic moment can be linear, quadratic or a combination of both, depending on the polarization of the incident X-ray beam and the direction of the magnetic moment. With a linearly polarized beam, the scattered intensity will have a purely quadratic dependence on the magnetic moment when the magnetic moment is parallel to the scattering plane. However, with the magnetic moment perpendicular to the scattering plane, there is also a linear component. This means that, when measuring the hysteresis with linear polarization during a hysteresis cycle, the intensity will be an even function of the applied field when the change in the magnetic moment (and field) is confined within the scattering plane but becomes more complicated when the magnetic moment is out of the scattering plane. Furthermore, with circular polarization, the dependence of the scattered intensity on the moment is a combination of linear and quadratic. With the moment parallel to the scattering plane, the linear component changes with the helicity of the incident beam. Surprisingly, in stark contrast to absorption studies, even when the magnetic moment is perpendicular to the scattering plane there is still a dependence on the moment with a linear component. This linear component is completely independent of the helicity of the beam, meaning that the hysteresis loops will not be inverted with helicity.

## Abstract 2

The combination of linear and non-linear dependence of intensity on a magnetic moment leads to different forms of reflectivity as a function of both polarization and the direction of the applied magnetic field. Using circular polarization, it is possible to measure the hysteresis when the field is perpendicular to the scattering plane (unlike X-ray absorption), but with no dependence on the helicity of the beam.

## 1. Introduction

The measurement of the reversal process in modern magnetic materials is essential for understanding their magnetic properties, particularly for materials used for spintronics (Kuroda *et al.*, 2005 ▸; Hirohata *et al.*, 2020 ▸; Khan *et al.*, 2021 ▸). There are many ways of measuring magnetic hysteresis. For example, bulk measurements can be achieved using the extremely sensitive technique of vibrating sample magnetometry (VSM), where the total moment is measured using pick-up coils (Hurt *et al.*, 2013 ▸). By combining this technique with the superconducting interference effect in the measurement circuit, it has become even more sensitive, now being able to measure tiny signals down to the 10^−7^ electromagnetic unit (e.m.u.) level. Surface sensitive measurements can be achieved using the magneto-optical Kerr effect (MOKE) technique pioneered by Bader *et al.* (Qiu & Bader, 1999 ▸, 2000 ▸; Osgood *et al.*, 1998 ▸). This technique has been developed enormously over the last few years to include microscopy measurements yielding important spatially resolved measurements on thin-film materials (Corb, 1988 ▸). This has proven to be an excellent technique for the visualization of magnetic domains in thin films (Hussain *et al.*, 2017 ▸) and other interesting nanosized structures (Stupakiewicz *et al.*, 2014 ▸). The surface sensitivity of MOKE measurements and VSM measurements, which measure the magnetization of the whole sample, have been combined to separate out surface and bulk effects [see, for example, Hendrych *et al.* (2014 ▸)].

For measuring microscopic effects more directly in field, neutron (Ankner & Felcher, 1999 ▸) and X-ray scattering (Hill & McMorrow, 1996 ▸; Hannon *et al.*, 1988 ▸) are both established probes. The more bulk-sensitive thermal neutron reflectometry technique contrasts starkly with the surface-sensitive X-ray scattering. For neutrons, the cross section for magnetic scattering, based on the internal flux density **B**, differs with the complexity of the X-ray interaction, particularly in the case of resonant scattering, which is related to the imbalance of spin-up and spin-down electrons at the Fermi energy. The resonant scattering offers a distinct advantage in element specificity as it is the only technique that is able to do this directly. Soft X-ray scattering has the further advantage of having a high sensitivity to magnetic structures with often very small micrometre-scale-sized beams.

Measurements of element-sensitive hysteresis loops with X-ray absorption have already yielded interesting results, *e.g.* on rare-earth transition-metal exchange springs (Stenning *et al.*, 2012 ▸, 2015 ▸) and in other thin-film systems (Chakarian *et al.*, 1995 ▸; Hellwig *et al.*, 2011 ▸). When using circular polarization, the absorption is proportional to the magnetic moment of the element projected onto the incident-beam direction; as a result, many studies measure the absorption directly to obtain the hysteresis curve, without the need to calculate the magnetic moment, *e.g.* see Hellwig *et al.* (2011 ▸). The values of atomic moments can be probed almost directly using the well known optical sum rules (Altarelli, 1993 ▸; van der Laan & Figueroa, 2014 ▸; Thole *et al.*, 1992 ▸).

Soft X-ray scattering and particularly reflectivity have also been used several times to monitor hysteresis behaviour. By keeping the detector fixed in scattering angle to monitor the intensity of a diffraction peak (Chmiel *et al.*, 2019 ▸) or reflectivity (Marrows *et al.*, 2005 ▸), much can be learned microscopically about the reversal process. In some exchange bias systems it is possible to measure the behaviour of the antiferromagnetic layers near the interface due to uncompensated moments that can be manipulated through the ferromagnetic layer using an applied magnetic field (Engel *et al.*, 2004 ▸; Fan *et al.*, 2022 ▸).

Notwithstanding the advantages of soft X-ray scattering in measuring the magnetic reversal process, some complexities need to be addressed so that the form of the scattering during the hysteresis cycle can be properly understood (Burn *et al.*, 2022 ▸). The main purpose of this work is to demonstrate the non-linear dependence of the scattered intensity. The shape of the reflectivity or scattering that results during a hysteresis cycle is therefore in general different to that of the magnetization during a hysteresis loop, and strongly dependent on geometry and the polarization state of the incident beam. This work also emphasizes the differences between absorption and scattering when using circular polarization. One of the highlights of this work is the demonstration that circularly polarized X-rays can be used to measure the magnetic reversal when the moment is changing perpendicular to the scattering plane. In this geometry, the form of the scattering during the hysteresis is independent of the helicity of the X-ray polarization.

The article is organized as follows. Section 2 begins with subsection 2.1 that explains the samples, their characterization and the main experimental setup of soft X-ray scattering. The next subsection 2.2 describes the theory of resonant magnetic soft X-ray reflectivity. The following subsection 2.3 describes the case of linear polarization, which in turn has subsections 2.3.1 and 2.3.2 describing the cases of moments in the scattering plane and out of the scattering plane, respectively, *i.e.* the effects of the hysteresis cycle on the reflectivity when the moments are forced to change parallel to the scattering plane and perpendicular to the scattering plane. Following this, in Section 2.4, the case of circular polarization is developed for moments changing in the scattering plane (Section 2.4.1) and perpendicular to the scattering plane (Section 2.4.2). Section 2.5 then contains some calculations from a thin film using an optical theory to support some of our findings. This is then followed by the *Conclusions* [Sec sec3].

## 2. Results and discussion

### 2.1. Experimental preliminaries

**Figure 1:**
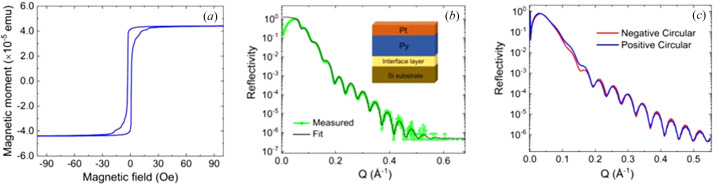
(*a*) A hysteresis loop of the thin film measured with a VSM. (*b*) Reflectivity of the thin film measured with X-rays at 8.05 keV (Cu *K*_α_). The reflectivity data are shown in green and the corresponding fit to the data is shown in black. For details of the fit, see the main text and Table 1 ▸. (*c*) The reflectivities at the Fe *L*_3_ edge with opposite helicities of circular polarization.

All measurements were carried out on a thin film of 10 nm of Py, an alloy of 80% Ni and 20% Fe, grown on Si(001) with a cap of 3 nm of Pt using DC magnetron sputtering. The hysteresis loop was measured with a Quantum Design vibrating magnetic sample magnetometer (MPMS3). This is shown in Fig. 1 ▸(*a*). The coercive field is less than 10 Oe, making it easy to saturate in our small magnet. In Fig. 1 ▸(*b*), X-ray reflectivity from our thin film is shown, which was measured at the Cu *K*α energy (8.05 keV). The fit to these data is achieved using the parameters in Table 1 ▸. The thicknesses match close to those expected from the growth rates. The root-mean-square roughnesses at the interfaces are all lower than 1 nm.

Soft X-ray reflectivity was measured on the I10 beamline at Diamond Light Source using the soft X-ray diffractometer RASOR (Beale *et al.*, 2010 ▸). The beamline consists of two helical undulators and employs a plane-grating monochromator design [we direct the interested reader to the work of Follath *et al.* (1998 ▸)]. The beamline focuses polarized light of 10^12^ photons s^−1^ at 780 eV with a bandpass of 0.1%. The light here is focused in a spot size of 200 µm × 200 µm (σ). The beamline is capable of linear or circular polarization (rates of circular polarization >99.8%). Reflectivities from the beamline are shown in Fig. 1 ▸. Each reflectivity was measured from our thin film at 707 eV with circularly polarized photons at opposite helicities. The difference between these two reflectivities arises because of the magnetization of the film (which was parallel to the film and in the scattering plane). The energy of 707 eV corresponds to the *L*_3_ resonance of Fe. All subsequent measurements in this article were carried out at this energy, where the photon scattering process is most sensitive to the magnetic moment of the Fe atoms. The measurements could also be done on the Ni *L*_3_ resonance, where the Ni atomic moments could be probed, but as the Ni and Fe atoms share the same magnetic environment the results and conclusions would be the same. The fringes in the reflectivity are the result of interference between the interfaces of the Py and Pt thin films. The magnetic field was applied using a small electromagnet attached to the sample holder. During application of the magnetic field, the sample and magnet were kept at room temperature by using a diffractometer flow cryostat with liquid nitrogen. A specially built sample holder enabled us to manually rotate the magnet so that fields could be applied parallel and perpendicular to the scattering plane.

### 2.2. Theory preliminaries

In the dipole approximation, the resonant-scattering form factor can be presented by the following expression (Hill & McMorrow, 1996 ▸; Hannon *et al.*, 1988 ▸) as 

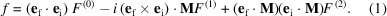

Here, **e**_i_ and **e**_f_ are directional vectors representing the incident and scattered polarization, respectively. Furthermore, **M** is the magnetic moment, and the coefficients *F* ^(0)^, *F* ^(1)^ and *F* ^(2)^ depend on the matrix elements involved in the resonant process. The first term is the charge scattering and the second term is the magnetic scattering to first order. The third term, which is second order in magnetization, is assumed to be negligible and will not be considered in the rest of this work.

We can now represent equation (1) in the following 2 × 2 matrix representation, where each element represents a particular incident and outgoing polarization change (ignoring the second-order term in the magnetic moment),

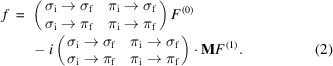

**Figure 2:**
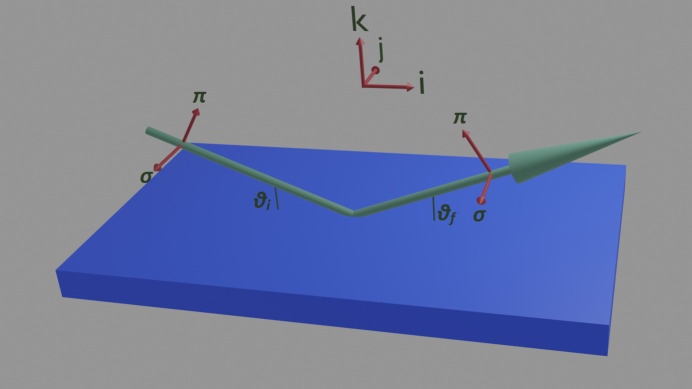
The frame of reference used for the calculations of polarization-dependent scattering. The Greek symbols π and σ refer to polarizations that are parallel or perpendicular to the scattering plane (the plane defined by the incoming and outgoing beam), respectively. The incident and outgoing angles are represented by θ_i_ and θ_f_, respectively. The suffixes i and f refer to the incident and scattered polarization, respectively. A right-handed set with unit vectors **i**, **j** and **k** is also shown for guidance.

Using the right-handed coordinate system in Fig. 2 ▸, assuming specular reflectivity (so that θ_i_ and θ_f_ are equal to θ), the first two terms in equation (1) become

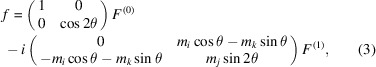

where 

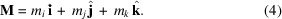

Here, the diagonal terms correspond to no rotation of the polarization between incident and scattered X-rays, whereas the off-diagonal terms correspond to rotations of the polarization.

We can now acknowledge that the charge scattering and magnetic scattering factors are complex quantities by making both *F* ^(0)^ and *F* ^(1)^ complex. This enables us to write equation (3) in the following form (see the supporting information for details), 

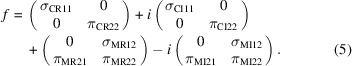

In the above we have made both the charge scattering and magnetic form factors complex to allow for the phase changes as the energy is adjusted in the vicinity of the resonance. Using equation (5), we can work out the intensity (*I* = f*f) for the different polarizations (circular and linear) with the magnetization in different directions (in the scattering plane and out of the scattering plane).

The calculation of magnetic reflectivity requires a knowledge of the values of the charge [ 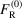 and 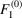 ] and magnetic [ 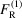 and 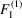 ] form factors. The real values are calculated using Kramers–Kronig (KK) transformation, assuming that the absorption spectra from the total electron yield provide the imaginary part of the form factor (see the supporting information for more details). All the calculations in this work have used these form factors, unless mentioned otherwise. Since the total electron yield is only sensitive to the first few nanometres of the thin film, it will be dominated by the Pt capping layer. The KK analysis will therefore be approximate and not yield accurate values of the scattering factors. Nevertheless, there is significant sensitivity of at least part of the NiFe film, as we clearly see the Fe resonance. Since the calculation of the exact reflectivity is not required, the resulting approximation to the scattering factors is appropriate enough here to demonstrate qualitatively the dependence of reflectivity during the hysteresis process.

### 2.3. Linear polarization

#### 2.3.1. Case 1: moments in the scattering plane

**Figure 3:**
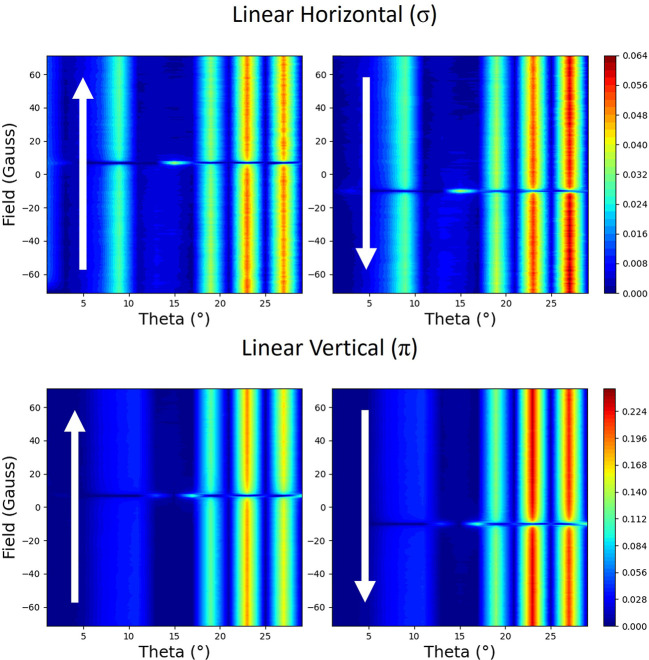
The measured reflectivity represented with colour maps plotted with applied magnetic field versus the angle of the incident beam to the surface (theta). The magnetic field is applied parallel to the scattering plane. Each polarization (linear horizontal σ and linear vertical π) is shown with two maps, which correspond to the two branches of the hysteresis curve. The direction of changing field is indicated by the arrows.

From setting the magnetic field to change along the scattering plane, parallel to the surface (parallel to **i** in Fig. 2 ▸), the resulting reflectivities are shown in Fig. 3 ▸. These were measured by positioning the detector at each point along the reflectivity and then cycling the magnetic field. At the top are shown reflectivities for the linear horizontal light (σ) for both branches of the hysteresis curve. At the bottom are shown reflectivities for the linear vertical light (π) for both branches. The fringes that are seen in the reflectivity in Fig. 1 ▸ are shown as vertical streaks here, in all of the plots. At the coercive field of roughly 10 Oe, there is a pronounced minimum along most of the reflectivity, although for some select parts, such as close to 15°, there is a maximum.

**Figure 4:**
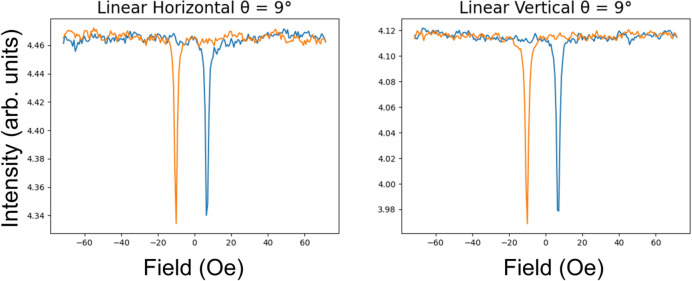
Hysteresis curves measured with linear light when the magnetic field is applied parallel to the scattering plane. These measurements were taken at an incidence angle of 9°. They are slices through the colour maps in Fig. 3 ▸ at this angle. The two colours represent each branch of the hysteresis. Blue represents increasing field and red represents decreasing field.

The hysteresis curves at 9° are shown in Fig. 4 ▸. These can be obtained by tracing vertical lines at 9° in Fig. 3 ▸. During the hysteresis cycle, the reflectivity is constant as a function of applied fields, apart from around the coercivity where there is a drop in intensity.

If the moments are kept in the scattering plane, only the off-diagonal terms contribute to the magnetic scattering, *i.e.* terms containing the factors π_MR22_ and π_MI22_ can be set to zero. This can be further simplified. In first-order electric dipole transitions, with the moments in the scattering plane, σ polarized light will give rise to π polarized magnetic scattering leading to the following equation (see the supporting information), 

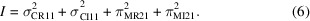

In the same way, for π incident polarization (where magnetically scattered light is all in the σ channel) we obtain 

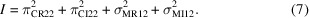

Here, in equations (6) and (7) we see that the intensity depends quadratically on π_MR21_, π_MI21_, σ_MR12_ and σ_MI12_. Since both of these terms are linear in magnetic moment, the intensity depends quadratically on magnetic moment as mentioned before. There are no interference terms between charge and magnetic scattering as it would not occur unless the anomalous scattering also rotated the polarization of the incident light. This has been seen in many materials, such as the normally forbidden [001] diffraction peak from Cu_2_OSeO_3_, *e.g.* Burn *et al.* (2021 ▸). At resonance, this peak appears and the polarization of the scattered light is rotated. In this case, the above expressions would need to be modified and would include linear interference terms where the outgoing magnetic and charge scattering mix and add together (see the supporting information). The strength of these linear terms would depend on the imaginary and real components of the charge scattering. Combined with the quadratic terms, the reflectivity measured during a hysteresis would have a different form as a function of the magnetization.

**Figure 5:**
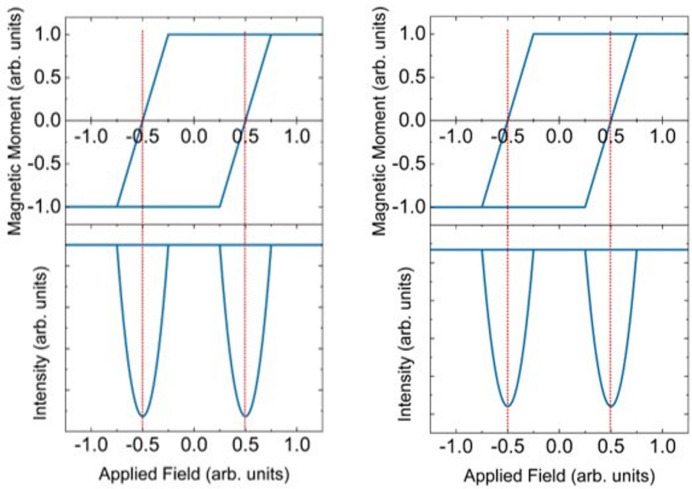
The behaviour of the scattering (bottom) is shown during the hysteresis cycle (top) when the applied field is parallel to the scattering plane (and parallel to the surface). This is shown when the incident polarization is perpendicular to the scattering plane (σ) on the left and parallel to the scattering plane on the right (π). The minimum in the reflectivity corresponds to the coercivity of the magnetic material. The clear quadratic dependence of the scattering on the magnetic moment is demonstrated.

To calculate the reflectivity using equation (7), we will use a simple hysteresis loop where the moment magnitude changes completely linearly in one dimension parallel to the sample surface with a coercivity of 0.5 arbitrary units. This is shown in the top plots of Fig. 5 ▸. Although unrealistic, its simplicity is sufficient to demonstrate the complexities of measuring the magnetic reversal process. In all models, the magnetization will change in one dimension, either parallel or perpendicular to the scattering plane (parallel to the surface), unless stated otherwise. There will be no component of the magnetic moment in the direction perpendicular to the surface (*i.e.* in the direction 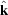 in Fig. 2 ▸) in any of the models.

The calculations of the scattering are shown in Fig. 5 ▸ during the hysteresis cycle. To calculate the scattering cross section, 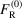 , 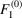 , 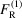 and 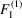 were set to values corresponding to those at 707 eV (8.50, 11.35, 2.56 and 3.83, respectively; see the supporting information) and the sample angle θ was set to 9°. The calculations shown in Fig. 5 ▸ are for both σ polarization using equation (6) and π polarization using equation (7).

The behaviour of the scattering with linear light is constant apart from two parabolic dips near the coercive fields. The reflectivity from our thin film will have a more complex behaviour in the vicinity of the coercive field than that predicted from our simple theory, as the moment will not change linearly with the applied field during the reversal process.

#### 2.3.2. Case 2: moments perpendicular to the scattering plane

**Figure 6:**
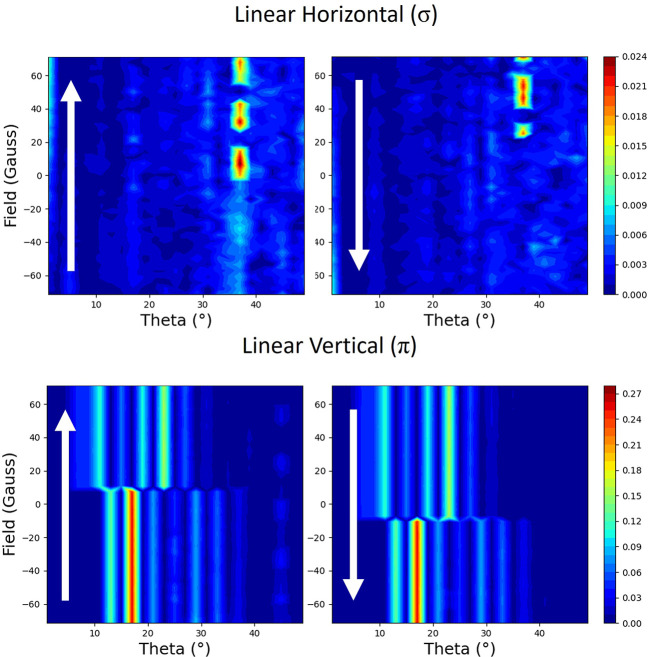
The measured reflectivity represented with colour maps plotted with applied magnetic field versus the angle of the incident beam to the surface (theta). The magnetic field is applied perpendicular to the scattering plane. Each polarization (linear horizontal σ and linear vertical π) is shown with two maps, which correspond to the two branches of the hysteresis curve. The direction of changing field is indicated by the arrows.

Measurements of the hysteresis cycle with linear light as the magnetic applied field is applied perpendicular to the scattering plane (parallel to **j** in Fig. 2 ▸) are shown in Fig. 6 ▸. Apart from some noise, there is no clear signal when measuring with σ polarized incident light. In contrast to this, when measuring with π polarized incident light, there is a clear hysteresis behaviour indicative of a strong linear dependence on the magnetic moment. Interestingly, the hysteresis curves switch signs depending on the incident angle.

**Figure 7:**
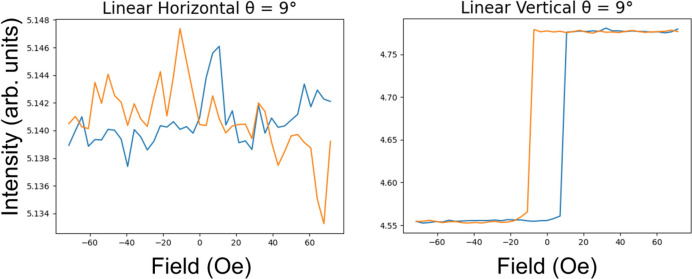
Hysteresis curves measured with linear light when the magnetic field is applied perpendicular to the scattering plane. These measurements were taken with an incidence angle of 9°. They are slices through the colour maps in Fig. 6 ▸ at this angle.

If we take a slice through Fig. 6 ▸ at 9° we obtain the hysteresis loops in Fig. 7 ▸. The reflectivity measured with σ polarized light is very noisy with no clear dependence on applied field, while that with π incident polarization shows a clear signal dependent on the magnetic moment of the film, as shown at many of the angles in Fig. 6 ▸.

**Figure 8:**
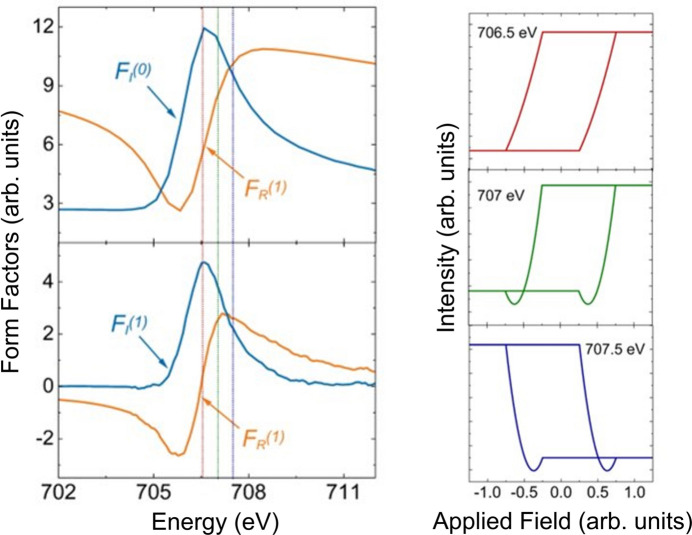
Calculations of reflectivity for π polarization when the magnetic moment is changing perpendicular to the scattering plane. On the left are the results of the form-factor calculations from the KK analysis. The calculations are carried out for the reflectivity using these form factors in equation (8) and using magnetic moments from the theoretical hysteresis curve shown in Fig. 5 ▸. Calculations for the reflectivity during a hysteresis cycle are shown for three energies: 706.5, 707 (at resonance) and 707.5 eV. The colours of the lines indicating the energies in the form-factor data correspond to the colours of the scattering calculation curves.

If the magnetic moment is perpendicular to the scattering plane, then only the one diagonal component is present in the magnetic part of the equation. There is therefore no rotation of the polarization when the X-rays are scattered by the magnetic ion, unlike the previous case in Section 2.3.1. The equation describing the intensity is now 

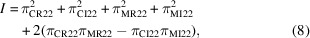

thus demonstrating that the dependence of the scattered intensity has both a linear and quadratic component. The relative sizes of the linear and quadratic dependences will depend on the sizes of the imaginary and real components of the charge scattering, which in turn will depend on the energy of the incident beam. By using the form factors calculated from the KK analysis (see the supporting information) we can look at the energy dependence, which will change the relative sizes of the quadratic and linear dependences to the magnetic moment. Results of these calculations using these form factors (and the behaviour of the magnetic moment dictated by the hysteresis loop in Fig. 5 ▸) are shown in Fig. 8 ▸. At 706.5 eV, the loop generated resembles the hysteresis. However, at 707 and 707.5 eV, a quadratic dependence on magnetic moment is clearly visible. In addition, the loop switches sense at 707.5 eV.

### 2.4. Circular polarization

#### 2.4.1. Case 3: moments in the scattering plane

**Figure 9:**
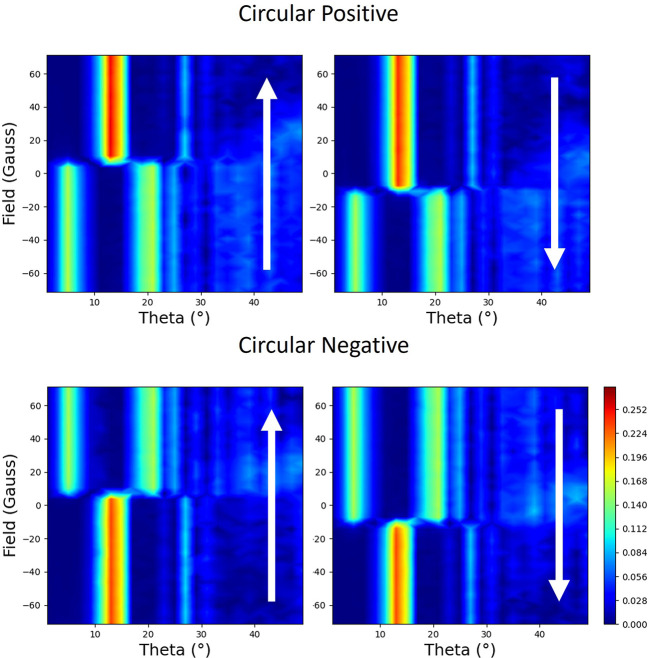
The measured reflectivity represented with colour maps plotted with applied magnetic field versus the angle of the incident beam to the surface (theta). The magnetic field is applied parallel to the scattering plane. Each polarization (opposite helicities of circular polarization designated as circular positive and circular negative) is shown with two maps, which correspond to the two branches of the hysteresis curve. The direction of changing field is indicated by the arrows.

**Figure 10:**
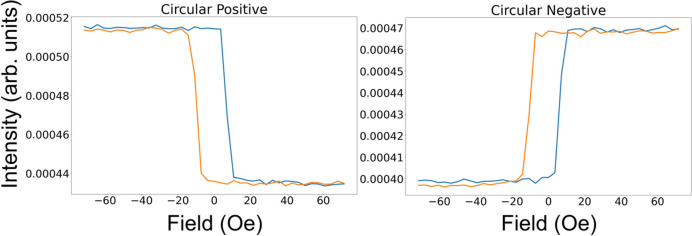
Reflectivity during the hysteresis cycle measured with circular light at 21° for both helicities. The field was applied parallel to the scattering plane.

Results for the magnetic field being applied parallel to the scattering plane during the hysteresis cycle are shown in Fig. 9 ▸. The reflectivity exhibits a strong linear behaviour with magnetic moment with circular polarization. This is particularly strong at the lower angles in the plots of Fig. 9 ▸. As in the linear vertical case with the applied field perpendicular to the scattering plane (see Fig. 6 ▸), the sense of the loop, *i.e.* which sign of the applied field has the larger reflectivity, is strongly dependent on the incident angle of the beam. In addition, the loops can be inverted when the helicity of the beam is changed, *i.e.* the light areas become dark and vice versa. Slices through these data at 21° are shown in Fig. 10 ▸, demonstrating the clear linear dependence on the magnetic moment. Also demonstrated is the dependence on the helicity of the circularly polarized beam.

**Figure 11:**
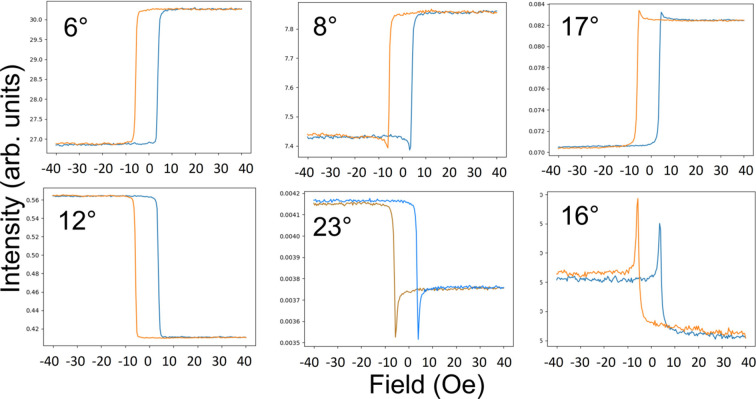
Reflectivity measured with negative circular light (all measured with identical helicity) at different sample angles during the hysteresis cycles.

To further examine some individual loops, slices were taken from the negative circular data, as shown in Fig. 11 ▸. Out of all the loops measured along the reflectivity profile, six types of hysteresis loops were found when measured with just one incident helicity of circular polarization. It is clear from the loops that there is a strong linear component of the reflectivity on the magnetic moment. In addition to this strong component, there are other components that either cause minima or maxima in the vicinity of the coercive field. As mentioned before, the sign of the loop depends on the angle θ of the incident beam.

In the case of circularly polarized X-rays, the amplitudes can be modelled as two orthogonal polarizations phase shifted by π/2 radians. In terms of σ and π polarization, this would take the form σ_*i*_ + *i*π_*i*_ and σ_*i*_ − *i*π_*i*_ for both helicities. When this phase difference is taken into account and the moments are kept entirely within the scattering plane, equation (5) becomes (see the supporting information) 

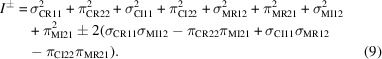

The first four terms (σ_CR11_, π_CR22_, σ_CI11_ and π_CI22_) are charge terms and do not change with magnetic field. The next four terms (σ_MR12_, π_MR21_, σ_MI12_ and π_MI21_) are quadratic in magnetic moment and are independent of the helicity of the beam. The last four terms (σ_CR11_σ_MI12_, π_CR22_π_MI21_, σ_CI11_σ_MR12_ and π_CI22_π_MR21_) are linear in magnetic moment. They are the result of interference between charge and magnetic scattering caused by a combination of circular polarization and the π/2 radian phase difference between the charge and magnetic form factors. The sign of these linear terms is dependent on the helicity.

**Figure 12:**
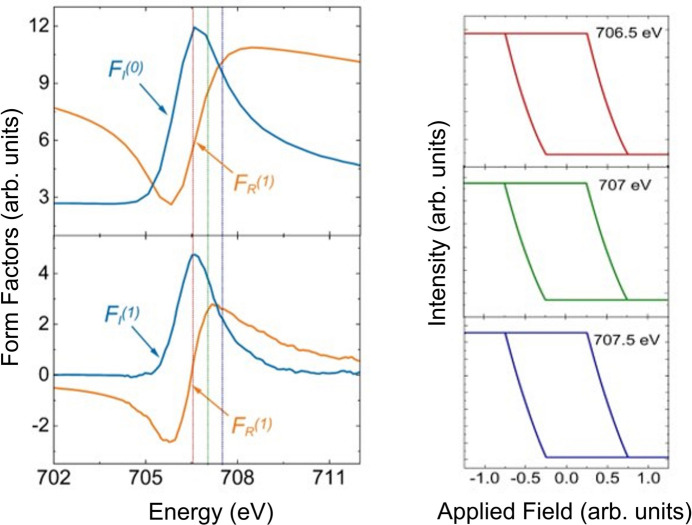
Calculations of the reflectivity during a hysteresis cycle are shown with circular polarization when the magnetic field is changing in the scattering plane. Three different loops are shown: the top one is at 706.5 eV, the middle one is at 707 eV (resonance) and the bottom one is at 707.5 eV. There is a strong linear component visible in all three hysteresis loops, which resembles the hysteresis loop but inverted.

Using the form factors at the resonance Fe *L*_3_ (707 eV), and also around the resonance (706.5 and 707.5 eV), we obtain the results given in Fig. 12 ▸ for one helicity. There is a strong linear component in all three loops as expected and non-linear effects are not visible.

If there are non-linear effects, they can be removed by measuring the scattering from two hysteresis loops with opposite helicities and then subtracting one from the other (see the supporting information).

The results shown in Fig. 11 ▸ are examples of the six qualitatively different hysteresis loops we found in the data from Fig. 9 ▸. They have all been measured with the same helicity of circular polarization. The top row contains a hysteresis loop with no significant quadratic dependence on moment (6°, on the left), one with non-linear effects appearing at the bottom (8°, in the centre) and one with the non-linear effects at the top (17°, on the right). The non-linear effects appear as minima or maxima centred around the coercive fields on the loop. In the bottom row, the other three hysteresis loops appear qualitatively as a reflection through the *y* (moment) axis at zero field of the three loops on the top row. Whilst our simple theory can describe the loops at 6° (mostly linear dependence on magnetic moment – see, for example, calculations in Fig. 12 ▸) and 8° (linear with significant quadratic dependence on magnetic moment), the other four loops need further explanation.

Thus far, the theory only changes the size of the magnetic moment along one direction: either in the scattering plane or perpendicular to the scattering plane. If the theory is extended so that a moment can rotate in the surface, which would perhaps be more realistic, then, as the moment points out of the scattering plane, the π_MR22_ and π_MI22_ components that have been neglected have to be taken into account. These terms will be maximum when the moment is actually perpendicular to the scattering plane. This means that as the moment rotates from parallel to the scattering plane to perpendicular then back to parallel these terms will increase from zero to a maximum (90°) to the scattering plane and back to zero as the moment rests back in the scattering plane.

To model a system where the moment rotates at a constant rate in the surface plane, we introduce a parameter that is the angle to the scattering plane. This parameter then increases from 0 to 180°, then back to 0°. This can be modelled by having the moment in the scattering plane depending on the cosine of this angle, whilst the moment out of the scattering plane depends on the sine of this angle (see the supporting information). This switching takes place during the same interval of applied magnetic field as the linear model as shown in Fig. 5 ▸, *i.e.* 0.25 to 0.75 arbitrary units and −0.25 to −0.75 arbitrary units. The theory represented in equation (9) also needs to be extended to include the effects of these out-of-plane moments. This is done by including the quadratic terms in π_MR22_ and π_MI22_, which are simply added to equation (9) (see the supporting information). Since our model is very simple and is only rotating the moments, we have assumed that the moments perpendicular to the scattering plane would sum to zero during the magnetic reversal process. This would mean that only the quadratic components of the moments would be finite and that all linear components are assumed to be zero.

**Figure 13:**
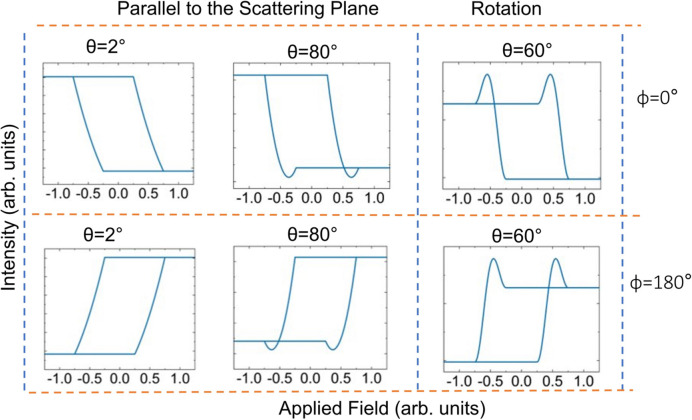
Calculations of the reflectivity at 707 eV with one helicity of circular polarization. Those on the left and in the middle were calculated with a moment varying linearly in-plane (see the hysteresis loop in Fig. 5 ▸) at θ incident angles of 2° and 80°. Those on the right were calculated with a reduced charge-scattering factor (see the main text) and by rotating the moment so that it is initially parallel to the scattering plane then rotates around to perpendicular to the scattering plane and back to parallel to the scattering plane. They were calculated at angles of incidence of 60° (as indicated in the figure). The top row was calculated with an added phase factor ϕ of 0° and the bottom row was calculated with a phase factor ϕ of 180° (see the main text for details).

With this model we should be able to simulate, at least qualitatively, the maximum around the coercive fields by taking into account both components of the magnetic moments parallel and perpendicular to the scattering plane. Calculations using the general case for circular polarization, *i.e.* equation (9) with the additional quadratic components of π_MR22_ and π_MI22_, have been done and are shown in Fig. 13 ▸. The top plots calculated at 2° and 80° were carried out with the moment in the scattering plane varying as demonstrated by the hysteresis loop in Fig. 5 ▸, *i.e.* the loop is linear around the coercive field. The scattering factors have been set to those corresponding to the Fe *L*_3_ resonance in our KK analysis. To make the quadratic effects visible, the calculations needed to be executed at 80°, but this could have also been done by artificially increasing the magnetic structure factors at a much lower angle. Here, the calculations have been carried out using equation (9) without the extra terms π_MR22_ and π_MI22_, since these are zero. These calculations simulate qualitatively the loops at 6° and 8°, respectively, in Fig. 11 ▸. On the right at the top of Fig. 13 ▸ is shown a calculation where the magnetic moment is rotating constantly from parallel to the scattering plane to perpendicular and back to parallel, from 0.25 to 0.75 arbitrary units of field. This exact process is then repeated when the moment is forced in the opposite direction from −0.25 to −0.75 arbitrary units of field. Due to the moment now being out of plane, π_MR22_ and π_MI22_ are now finite and need to be taken into account. The results of the calculations are dependent on incident angle θ. At 60°, strong maxima are seen around the coercive field if the size of the charge structure factor is reduced by a factor of ten. If we do not decrease the charge scattering relative to the magnetic scattering, these effects are too small to be visible. This indicates that these effects only occur at points along the reflectivity where the charge form factors are small compared with the magnetic form factors. This simple atomic model is only qualitative in order to demonstrate the main features of the scattering. The angular dependence of the features from the thin film in the experiment shown in Fig. 11 ▸ is very different to that from our simple calculation involving a smooth rotation of all of the moments.

So, qualitatively, three of the experimental loops in Fig. 11 ▸ have been described. It is now possible to describe the loops that look like the reflectivity hysteresis curves from conventional magnetometry, *i.e.* have a strong linear component in magnetic moment (such as at a θ of 6°). It is also possible to qualitatively explain the loops with minima around the coercive fields (such as at a θ of 8°). By taking into account moments perpendicular to the scattering plane, we are also able to account for maxima around the coercive fields (*e.g.* at 16°). However, there are still three loops that have switched sense, which have not been described. All these loops were measured with the same helicity (it is understood that switching the helicity can also switch the sense of the loops).

To switch the sense of the loops, a phase needs to be introduced. In scattering, the exact phase is lost, but since there are terms linearly dependent on magnetic moment these terms could be reversed by the effect of a phase. This can be done by introducing the phase in equation (9) (see the supporting information). The origin of this phase, designated as ϕ, will be commented on later. Its effect is introduced as a cosine, which only affects the terms that are linear in magnetic moment. This is justified in our qualitative model since the sign change resulting from this phase would be lost in the quadratic terms.

For the reflectivity curves we have already calculated in the top of Fig. 13 ▸, the phase ϕ is effectively set to 0°. If we set the phase ϕ to 180°, we can reverse the signs of the linear terms in equation (9). This is shown for the bottom-left and bottom-middle plots, which are the equivalent of the top-left and top-middle plots at incoming angles of 2° and 80° but with the phase ϕ set to 180°. By using the phase factor in equation (9), but including the additional quadratic components of π_MR22_ and π_MI22_, the sense of the linear components of the hysteresis loop in the top right of Fig. 13 ▸ can also be changed, as can be seen in the calculated curve underneath it.

With our simple atomic model, we have thus been able to qualitatively explain all the main features of the six families of loops in Fig. 11 ▸. This has been done by utilizing the relative dependence of linear and quadratic terms as a function of angle, invoking the dependence of the out-of-plane component for more complex magnetic reversal, and introducing a simple phase factor that will only influence the linear magnetic terms.

The non-linear effects in the reflectivity hysteresis loops become very strong when the charge scattering is weaker. The hysteresis loops measured at 16°, 17° and 23° all occur at or near minima in the reflectivity (see Fig. 9 ▸), whereas those measured at 6° and 12° occur where the charge scattering is relatively stronger. The loop measured at 8°, which also shows a lot of non-linearity, has very strong charge scattering, but there is a slight minimum occurring at the reflectivity. If the magnetic structure of the film is different to that of the chemical structure, as in the case of the heterostructure measured here (Pt is maybe slightly magnetized at the interface but our experiment would not be sensitive to this), then at angles where the magnetic scattering is relatively weak, the linear effects, which depend on both the magnetic and charge scattering being significant, will also be weak. This could lead to non-linear components of the scattering being more prevalent.

#### 2.4.2. Case 4: moments perpendicular to the scattering plane

**Figure 14:**
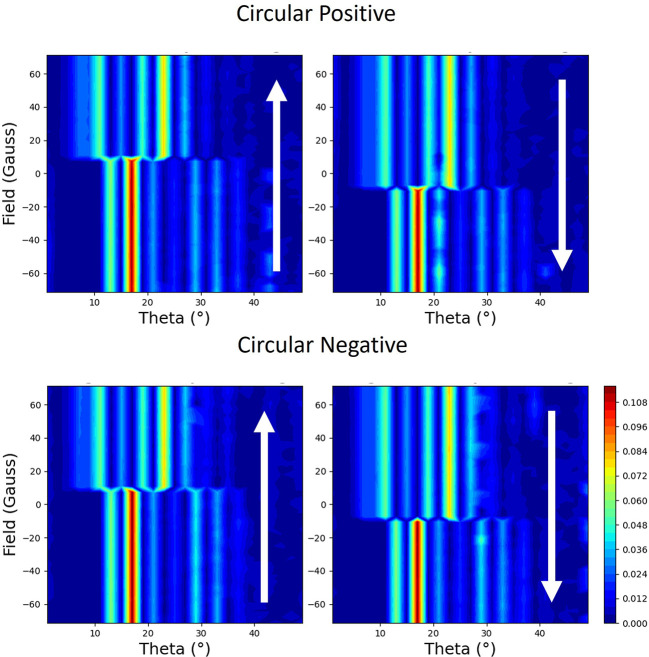
The measured reflectivity represented with colour maps plotted with applied magnetic field versus the angle of the incident beam to the surface (theta). The magnetic field is applied perpendicular to the scattering plane. Each polarization (opposite helicities of circular polarization designated as circular positive and circular negative) is shown with two maps, which correspond to the two branches of the hysteresis curve. The direction of changing field is indicated by the arrows.

Reflectivity measured during the hysteresis cycle with the applied field perpendicular to the scattering plane is shown in Fig. 14 ▸. If one looks at the top two plots, which were taken for the same handedness of circular light, there is a clear linear dependence on the magnetic moment. Since the applied field is now driving the magnetization perpendicular to the scattering plane, this seems to be counterintuitive. If the same experiment was done by measuring the absorption, rather than scattering, you would not have any sensitivity to the moments in this direction. This behaviour is similar to the case where the applied field is in the scattering plane in Section 2.4.1. In stark contrast, the reflectivity loops measured with the opposite helicity of circular polarization, shown in the bottom of Fig. 14 ▸, demonstrate that this scattering is independent of helicity.

**Figure 15:**
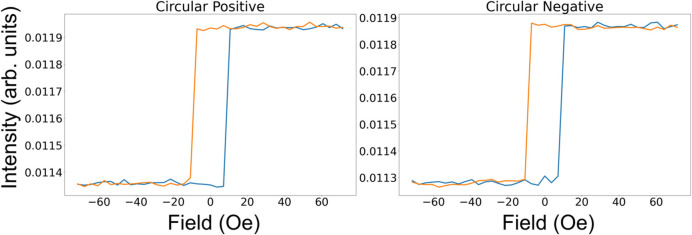
Reflectivity during the hysteresis cycle measured with circular light at 20° for both helicities. The field was applied perpendicular to the scattering plane.

Slices from the data in Fig. 14 ▸ at 20° are shown in Fig. 15 ▸. Here, the two square hysteresis loops clearly demonstrate that there is a strong linear dependence on the moment perpendicular to the scattering plane but that this effect is independent of the helicity of the circularly polarized beam.

For moments perpendicular to the scattering plane, the general equation (5), for both helicities, simplifies to (see the supporting information)

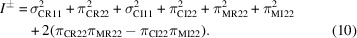

The equations for both helicities are identical, which completely corroborates the data in Figs. 14 ▸ and 15 ▸, where the data from the two helicities are also identical. Furthermore, equation (10) is identical to that of equation (8), apart from the charge terms σ_CR11_ and σ_CI11_ (which do not change with magnetic field).

**Figure 16:**
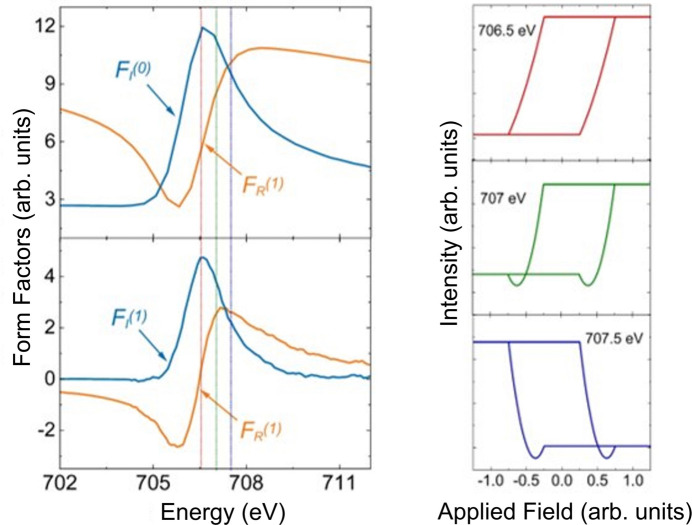
Calculations of reflectivity during the hysteresis cycle measured with circular light at 9° using our simple model at energies of 706.5, 707 and 707.5 eV. The form factors are shown on the left.

Fig. 16 ▸ represents calculations using equation (10) at different energies. The form factors are shown on the left. Whilst the loop at 706.5 eV looks mostly linear, quadratic effects are evident in 707 and 707.5 eV. The loop at 707.5 eV is also in the opposite sense to the other two loops. It is no accident that Fig. 16 ▸ resembles that of Fig. 8 ▸. Since in both cases it is only the π polarization that interacts with the magnetic moment, this is not surprising.

In the same way as for circular polarization, but with the magnetic field applied parallel to the scattering plane, there will be non-linear effects in the hysteresis curves. These will have the same origin, which has already been discussed in detail.

### 2.5. Optical modelling of the thin film

**Figure 17:**
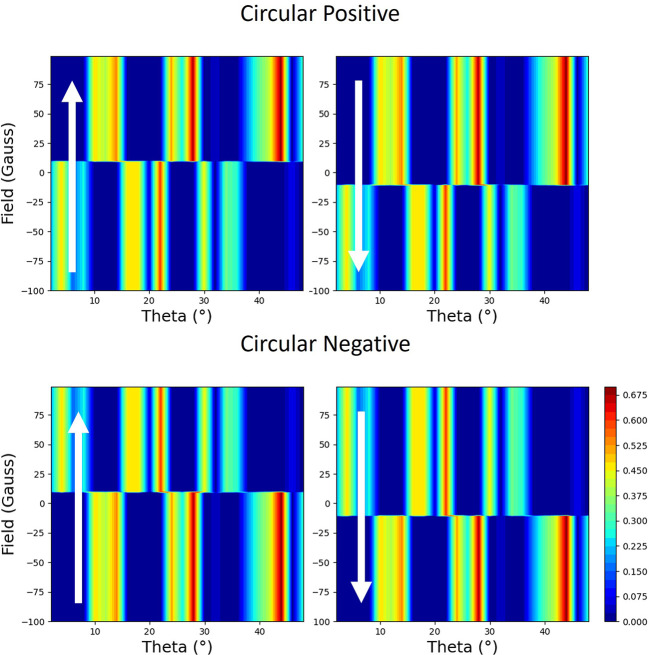
Simulated two-dimensional plots of the normalized reflectivity with opposite helicities of circular polarization during the hysteresis loop from 10 nm thin film of Py capped with 3 nm of Pt on an Si substrate. The field is parallel to the scattering plane. The magnetic field is plotted against the sample angle (theta), with values of normalized magnetic reflectivity represented by the colour map. The direction of changing field is indicated by the arrows.

The calculations so far have been carried out using a simplified model, as a demonstration that the general shapes of the loops can be calculated and described qualitatively. By using software (Macke *et al.*, 2014 ▸) based on an optical theory, which can be used to simulate scattering from thin films and multilayers (Zak *et al.*, 1990*a* ▸,*b* ▸, 1991 ▸), we have modelled the reflectivity from our thin film. Although the theory is based on classical optical theory rather than a quantum mechanical approach (Hannon *et al.*, 1988 ▸), it reproduces the basic trends. It can be shown that the optical theory is symmetrically equivalent to the atomic theory represented by equation (1) to the first order. In Figs. 17 ▸ and 18 ▸, we have plotted the results from the model for circular polarization with the field applied parallel and perpendicular to the scattering plane, respectively. Whilst the calculation is missing some of the finer details, it does show that the loop has a strong linear component, which switches sign (loop is reflected through the vertical moment axis) with helicity in the parallel case but does not switch sign when the field is perpendicular.

**Figure 19:**
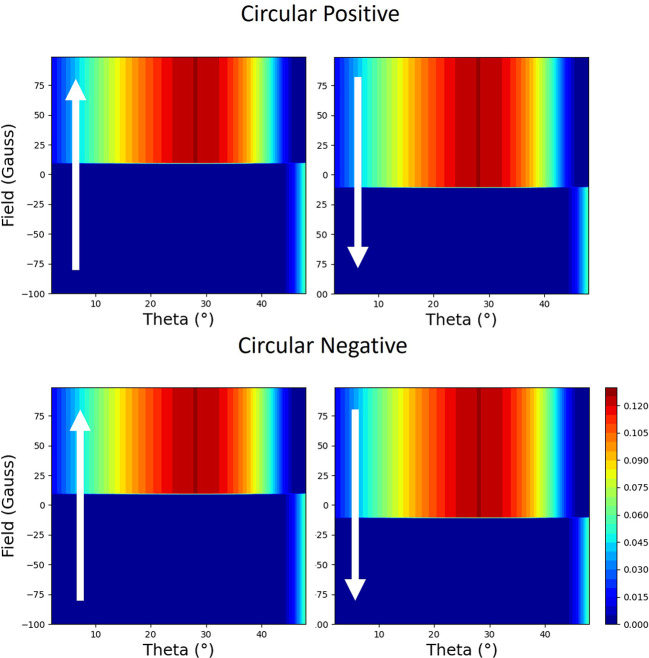
Simulated two-dimensional plots of the normalized reflectivity with opposite helicities of circular polarization during the hysteresis loop from a semi-infinite film of Py without a Pt capping layer or an Si substrate. The field is perpendicular to the scattering plane. The magnetic field is plotted against the sample angle (theta), with values of normalized magnetic reflectivity represented by the colour map. The direction of changing field is indicated by the arrows.

Also using the same optical theory, we tested the idea that the switching of the loops, which depends on the angle θ, is due to interference with the substrate and the Pt capping layer. This was achieved by removing them from the calculation and making the Py semi-infinite. This suppresses the effect of all of the interfaces from the calculation. The result is shown in Fig. 19 ▸. Here, there is no change in the sense of hysteresis up until θ = 45°, where, as was stated earlier, there is a change in the sign given by the interference of charge and magnetic terms in equation (1) for π to π scattering, *i.e.* the magnetic 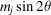 and charge 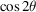 terms in equation (3). This causes the charge scattering to change sign at θ = 45°.

## 3. Conclusions

In this article, we have taken some simple examples and calculated the reflected intensity during hysteresis cycles under various polarizations and magnetization directions. The results highlight possible problems with interpretation owing to the non-linearity of the scattering dependence on the magnetic moment.

With linear polarization where the moment is within the scattering plane, there will be no interference between the charge and magnetic scattering. This is the case for both linear-vertical and linear-horizontal polarization, in and out of the scattering plane. However, if the charge scattering causes the polarization to rotate (anisotropic anomalous scattering), there will be interference between the magnetic and charge scattering. In the absence of interference, the dependence on the moment will be purely quadratic. When the moment is changing perpendicular to the scattering plane, in addition to this quadratic contribution there is an interference term, which depends linearly on the magnetic moment and both the imaginary and real parts of the charge form factor.

With circular polarization and the magnetization in the scattering plane, due to the π/2 phase difference between the real part of the charge scattering and the magnetic scattering, there is a strong interference term with a linear dependence on magnetization. This flips with the helicity. When the magnetization is changing perpendicular to the scattering plane, there is also a strong linear dependence. This arises due to the interference between the σ components of the charge and magnetic scattering. Rather counter-intuitively, this dependence does not change with the helicity of the incident beam. As well as the linear component, there is also a quadratic dependence. With the magnetization within the scattering plane, this quadratic dependence can be filtered out by subtracting two hysteresis loops taken with opposite helicities (dichroism), which will recover the original form of the hysteresis loop, *i.e.* the dichroism has a linear dependence on the magnetic moment. Finally, when the magnetic reversal is confined within the scattering plane, we classify six different shapes of hysteresis curves, which can be explained by relative contributions of the linear and non-linear dependence on magnetic moment, dependence on moments out of the scattering plane, and interference at the interfaces.

## 4. Related literature

The following references are only cited in the supporting information for this article: Brück (2009 ▸) and Henke *et al.* (1993 ▸).

## Supplementary Material

Supporting information. DOI: 10.1107/S160057752400119X/ye5035sup1.pdf

## Figures and Tables

**Figure 18:**
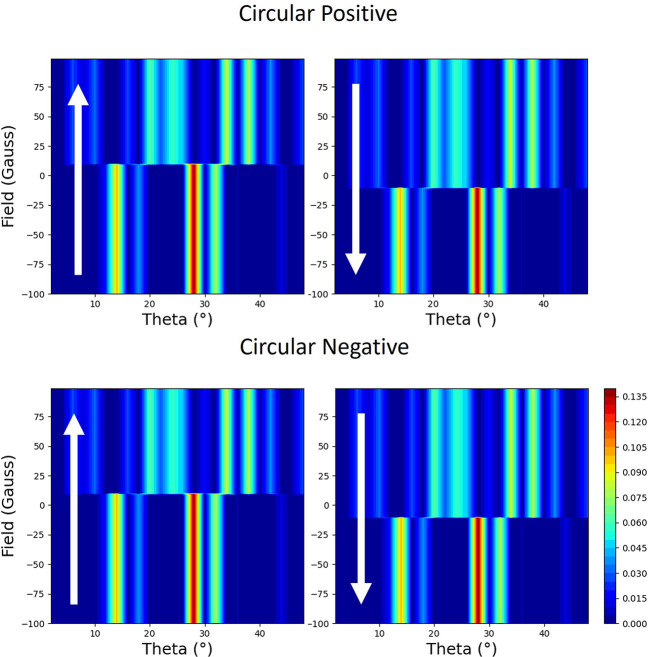
Simulated two-dimensional plots of the normalized reflectivity with opposite helicities of circular polarization during the hysteresis loop from 10 nm thin film of Py capped with 3 nm of Pt on an Si substrate. The field is perpendicular to the scattering plane. The magnetic field is plotted against the sample angle (theta), with values of normalized magnetic reflectivity represented by the colour map. The direction of changing field is indicated by the arrows.

**Table 1:** The parameters used to calculate the reflectivity in Fig. 1 ▸ for the Py and Pt thin film

Layer	Thickness (nm)	Roughness (nm)
Pt	3.2	0.49
Py	10.6	0.78
Native oxide	1.5	0.39
