# Supplementary material for: Measuring magnetic hysteresis curves with polarized soft X-ray resonant reflectivity
Source: J Synchrotron Radiat. 2024 Apr 10;31(Pt 3):493–507. doi: 10.1107/S160057752400119X (PMC11075705; doi:10.1107/S160057752400119X)
Supplement: Supplementary file 1 [file s-31-00493-sup1.pdf]

JOURNAL OF SYNCHROTRON RADIATION

Volume 31 (2024)

Supporting information for article:

Measuring magnetic hysteresis curves with polarized soft X-ray resonant reflectivity

R. Fan, Kiranjot, R. O. M. Aboljadayel, Kalel Alsaeed, Peter Bencok, D. M. Burn, A. T. Hindmarch and Paul Steadman

Measuring Magnetic Hysteresis Curves with Polarised Soft X-ray Resonant Reflectivity: Supplementary Material

R Fan,[1] Kiranjot,[1] R. O. M. Aboljadayel,[1] Kalel Alsaeed,[2]
Peter Bencok,[1] D. M. Burn,[1] A. T. Hindmarch[2] and Paul Steadman [1]*

[1] *Diamond Light Source, Harwell Science and Innovation Campus, Didcot, Oxon OX11 0DE United Kingdom, and* [2] *University of Durham, DH1 3LE United Kingdom. E-mail: Paul.Steadman@diamond.ac.uk*

1. General Theory

In the dipole approximation the resonant scattering form factor can be presented in the following expression (we direct the interested reader to references (Hill *et al.*, 1996; Hannon *et al.*, 1988) for more details) as

$$f = (\mathbf{e}_f \cdot \mathbf{e}_i)\ F^{(0)} - i(\mathbf{e}_f \times \mathbf{e}_i) \cdot \mathbf{M} F^{(1)} + (\mathbf{e}_f \cdot \mathbf{M})(\mathbf{e}_i \cdot \mathbf{M}) F^{(2)}. \quad (1)$$

Here $\mathbf{e}_i$ and $\mathbf{e}_f$ are directional vectors representing the incident and scattered polarisation respectively, $\mathbf{M}$ is the magnetic moment and the coefficients $F^{(0)}$, $F^{(1)}$ and $F^{(2)}$ depend on the matrix elements involved in the resonant process. The discussion of these coefficients is out of the scope of this work and not necessary for our conclusions.

The first term is the resonant charge scattering with a polarisation dependence that depends on the angle of the scattered beam relative to the incident beam. This has the same polarisation dependence as the non-resonant Thompson scattering. The

second term has a first order dependence between the projection of the polarisation vector product and the magnetic moment. The third term is second order in magnetic moment and therefore will be assumed to be negligible in this work.

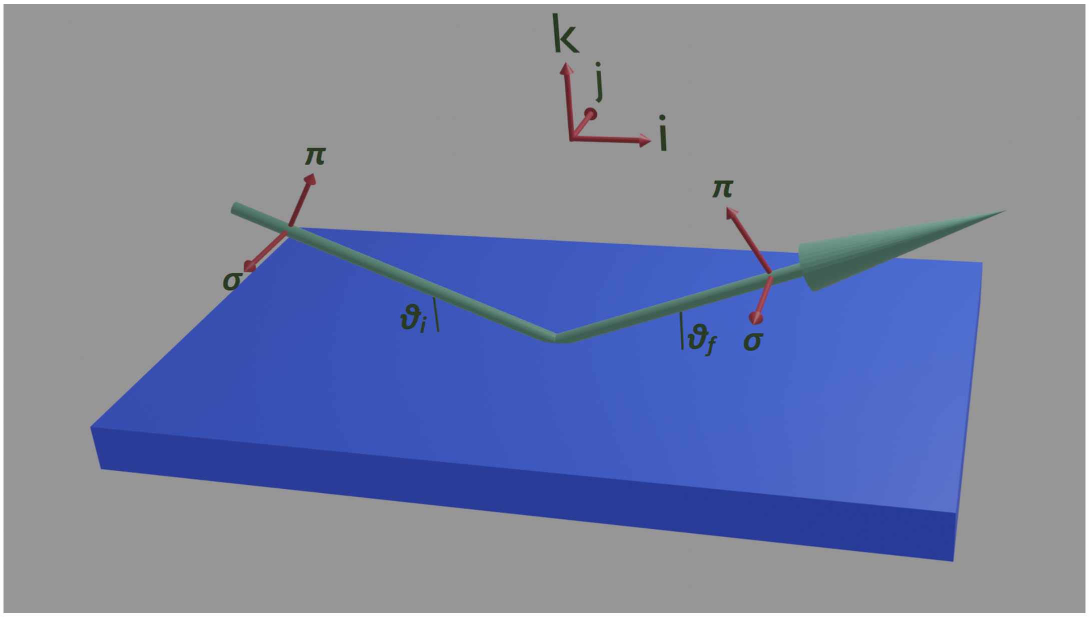

Fig. 1. The frame of reference used for the calculations of polarisation dependent scattering. The Greek symbols π and σ refer to polarisation that are parallel or perpendicular to the scattering plane (plane defined by incoming and outgoing beam) respectively. The suffixes i and f refer to the incident and scattered polarisation respectiely. The incident and outgoing angles are represented by θ_i and θ_f respectively. A right-handed set with unit vectors i, j and k is shown on the right for reference.

We will now use a simple reference frame as shown in Fig 1 based on a right-handed set. Fig 1 shows the incident and outgoing beams. The general polarisation state of the incoming and outgoing beams i.e. $\mathbf{e}_i$ and $\mathbf{e}_f$ can be separated into components with directions relative to the scattering plane. The polarisations will from now on in this work be represented by the Greek symbols π and σ which refer to polarisations parallel and perpendicular to the scattering plane respectively. The suffixes i and f refer to the incoming and outgoing beam with θ being both the incoming and outgoing

angle. In this frame of reference the polarisations can be represented in terms of the Cartesian unit vector system by

$$\boldsymbol{\pi_i} = \pi_i \sin(\theta_i)\mathbf{i} + \pi_i \cos(\theta_i)\mathbf{k}, \tag{2}$$

$$\boldsymbol{\sigma_i} = \sigma_i \mathbf{j}, \tag{3}$$

$$\boldsymbol{\pi_f} = -\pi_f \sin(\theta_f)\mathbf{i} + \pi_f \cos(\theta_f)\mathbf{k}, \tag{4}$$

and

$$\boldsymbol{\sigma_f} = \sigma_f \mathbf{j}. \tag{5}$$

The second term in Equation 1 is a result of the reduction in symmetry from the magnetic moment. The vector nature of the magnetic moment on the atoms can change the polarisation of the outgoing wave relative to the incoming wave. The first term in Equation 1 i.e. the charge scattering does not rotate the plane of polarisation. This is not the case in general since resonant charge scattering can introduce symmetry breaking because of the shapes of the intermediate state orbitals during the resonant process. In this work we assume that the charge scattering does not rotate the polarisation. The following 2x2 matrix representation where each element represents a particular incident and outgoing polarisation will now be defined (ignoring the second order term in magnetic moment):

$$f = \begin{pmatrix} \sigma_i \to \sigma_f & \pi_i \to \sigma_f \\ \sigma_i \to \pi_f & \pi_i \to \pi_f \end{pmatrix} F^{(0)} - i \begin{pmatrix} \sigma_i \to \sigma_f & \pi_i \to \sigma_f \\ \sigma_i \to \pi_f & \pi_i \to \pi_f \end{pmatrix} \cdot \mathbf{M} F^{(1)}. \tag{6}$$

Using the definitions in Equations (2- 5), assuming specular reflectivity (so that θ_i and θ_f are equal to θ) the first two terms in Equation 1 become:

$$f = \begin{pmatrix} 1 & 0 \\ 0 & \cos 2\theta \end{pmatrix} F^{(0)} - i \begin{pmatrix} 0 & m_i \cos\theta - m_k \sin\theta \\ -m_i \cos\theta - m_k \sin\theta & m_j \sin 2\theta \end{pmatrix} F^{(1)}, \tag{7}$$

where

$$\mathbf{M} = m_i\hat{\mathbf{i}} + m_j\hat{\mathbf{j}} + m_k\hat{\mathbf{k}}. \tag{8}$$

Equation 7 requires some explanation. The first term has only diagonal components meaning that there is no rotation of the polarisation due to the scalar nature of the charge scattering assumed in this work. Since the π polarisation is in the scattering plane the $\pi_i \rightarrow \pi_f$ term has an angular dependence due to the angle of the detector with respect to the incident beam. The σ term will have no such dependence as it is perpendicular to the scattering plane and independent of the detector angle.

The second term contains one diagonal term and two off-diagonal terms. There is no $\sigma_i \rightarrow \sigma_f$ term but there is a $\pi_i \rightarrow \pi_f$ term that has a sinusoidal dependence on twice the scattering angle (c.f. the cosinusoidal dependence of the charge scattering). In addition this depends on the magnitude of the magnetic moment (m_j) perpendicular to the scattering plane. This term corresponds to an exchange of angular momentum perpendicular to the scattering plane caused by a combination of the π polarisation and the difference between the directions of the incoming and outgoing wave vectors. This dependence will be zero at 0 $^\circ$ and the larger the difference the larger the exchange of momentum hence the sinusoidal dependence. This dependence does not exist for the σ polarisation as the X-ray amplitude is perpendicular to the scattering plane. There is therefore no exchange of angular momentum so, to first order in the dipole approximation, no dependence on the magnetic moment.

The off-diagonal terms in Equations 6 and 7 correspond to a rotation of the polarization, which demonstrates the exchange of angular momentum from the moment to that of the X-ray polarization. The $\sigma_i \rightarrow \pi_f$ term depends on the component of

the magnetic moment in the direction of the incident beam i.e. $m_i\cos\theta\ - m_k\sin\theta$ as in circular magnetic dichroism. This compares to the $\pi_i \rightarrow \sigma_f$ term depends on the component of the moment in the negative to the direction of the outgoing beam i.e. -$m_i\cos\theta\ - m_k\sin\theta$. On examination of all the terms in Equation 6 it can be seen that in an instrument that provides an X-ray beam with a well-defined polarisation and a polarisation analyser the magnetic and charge scattering can be completely separated.

At low angles the dominant contribution to the magnetic scattering comes from the off-diagonal terms (assuming that the $\pi_i \rightarrow \pi_f$ is small). In any case if the magnetic moments are completely in the scattering plane, then there are only the two off-diagonal terms. Since the charge scattering has no off diagonal terms in this simple example then there can be no interference between charge and magnetic scattering with linear polarisation.

We now make the following definitions and acknowledge that the charge scattering and magnetic scattering factor is a complex quantity by making both $F^{(0)}$ and $F^{(1)}$ complex,

$$\begin{aligned} F^{(0)} &= F_R^{(0)} + iF_I^{(0)} \\ F^{(1)} &= F_R^{(1)} + iF_I^{(1)} \end{aligned} \tag{9}$$

We then define

$$\sigma_{CR11} = F_R^{(0)}, \tag{10}$$

$$\pi_{CR22} = \cos(2\theta)F_R^{(0)}, \tag{11}$$

$$\sigma_{CI11} = F_I^{(0)}, \tag{12}$$

$$\pi_{CI22} = \cos(2\theta)F_I^{(0)}, \tag{13}$$

$$\sigma_{MR12} = (m_i \cos\theta - m_k \sin\theta) F_I^{(1)}, \tag{14}$$

$$\pi_{MR21} = (-m_i \cos\theta - m_k \sin\theta) F_I^{(1)}, \tag{15}$$

$$\pi_{MR22} = (m_j \sin 2\theta) F_I^{(1)}. \tag{16}$$

$$\sigma_{MI12} = (m_i \cos\theta - m_k \sin\theta) F_R^{(1)}, \tag{17}$$

$$\pi_{MI21} = (-m_i \cos\theta - m_k \sin\theta) F_R^{(1)}, \tag{18}$$

and

$$\pi_{MI22} = (m_j \sin 2\theta) F_R^{(1)}. \tag{19}$$

This enables us to write Equation 7 as

$$f = \begin{pmatrix} \sigma_{CR11} & 0 \\ 0 & \pi_{CR22} \end{pmatrix} + i \begin{pmatrix} \sigma_{CI11} & 0 \\ 0 & \pi_{CI22} \end{pmatrix} + \begin{pmatrix} 0 & \sigma_{MR12} \\ \pi_{MR21} & \pi_{MR22} \end{pmatrix} - i \begin{pmatrix} 0 & \sigma_{MI12} \\ \pi_{MI21} & \pi_{MI22} \end{pmatrix}. \tag{20}$$

In the above we have made both the charge scattering and magnetic form factors complex to allow for the phase changes as the energy is adjusted in the vicinity of the resonance. Using Equation 20 with the definitions in Equations (9 - 16) we work out the cases for the intensity ($I = f^* f$) for different polarisations (circular and linear) with the magnetisation in different directions (in the scattering plane and out-of-the scattering plane).

The calculation of magnetic reflectivity requires a knowledge of the values of charge ($F_R^{(0)}$, $F_I^{(0)}$) and magnetic ($F_R^{(1)}$ and $F_I^{(1)}$) form factors. To calculate the scattering

factors we measured absorption and dichroism of the film in total electron yield mode at 45° incident angle using both helicities of circular polarisation. The measured spectra for both polarisations were averaged and then fitted to the imaginary part of charge scattering factors in the Henke tables (HENKE *et al.*, 1993) in the 30eV to 30keV energy range. The Kramers-Kronig relations were then used to calculate the real part of the charge scattering factors. Similarly, the difference in imaginary scattering factors (which is the imaginary part of the magnetic scattering factors) was taken as input and the Kramers-Kronig relations were used to calculate the real part of the magnetic scattering factors (Brück, 2009). This measurement of absorption by total electron yield is not sensitive to the whole film and will be dominated by the Pt capping layer. There is, however, significant sensitivity to the FeNi layer due to the obvious presence of the Fe resonance in the data. In addition whilst the Henke table data, which is used to provide data away from the vicinity of the resonance, do not provide polarisation dependent intensities, at soft X-ray energies, the polarisation dependence is negligible here (polarisation dependence only occurs on resonance). The method will not necessarily yield accurate values for the scattering factors but give rough estimates which can be used in our calculations as a qualitative demonstration of the changes in reflectivity during the magnetic reversal process.

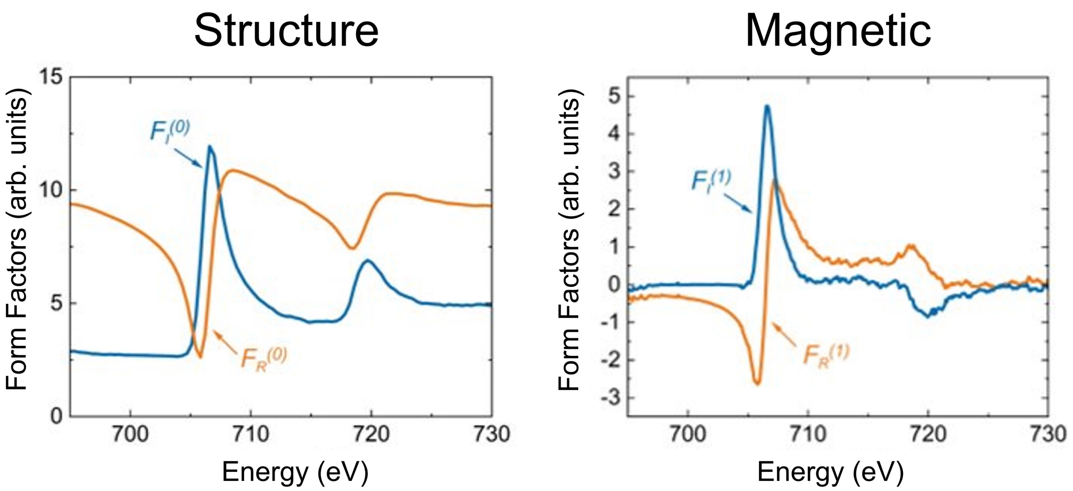

Fig. 2. The real and imaginary parts of the structural and magnetic form factor extracted from a reflectivity spectrum taken over both the L_2 and L_3 edges using the Kramers-Kronig transformation.

1.1. *Linear Polarisation*

1.1.1. Case 1: Moments in the Scattering Plane By taking the relevant terms in Equation 20 and finding the intensity ($I = f^* f$) the results can be calculated for the general case of linear polarised light scattered from a magnetic moment using the following equation.

$$I = \sigma^2_{CR11} + \pi^2_{CR22} + \sigma^2_{CI11} + \pi^2_{CI22} + \sigma^2_{MR12} + \pi^2_{MR21} + \sigma^2_{MI12} + \sigma^2_{MI21} + \pi^2_{MR22} + \pi^2_{MI22} + 2\pi_{CR22}\pi_{MR22} - 2\pi_{CI22}\pi_{MI22} \tag{21}$$

If the moments are kept in the scattering plane only the off-diagonal terms which contribute to the magnetic scattering i.e. so that terms containing the factor π_{MR22} and π_{MI22} can be set to zero. This can be further simplified. In first order electric dipole transitions, with the moments in the scattering plane, σ polarised light will give rise to π polarised magnetic scattering leading to the following equation.

$$I = \sigma^2_{CR11} + \sigma^2_{CI11} + \pi^2_{MR21} + \pi^2_{MI21} \tag{22}$$

In the same way for π incident polarisation (where magnetically scattered light is all in the σ channel) we obtain

$$I = \pi^2_{CR22} + \pi^2_{CI22} + \sigma^2_{MR12} + \sigma^2_{MI12} \tag{23}$$

1.1.2. Case 2: Moments Perpendicular to the Scattering Plane If the magnetic moment is perpendicular to the scattering plane, then only the one diagonal component is present in the magnetic part of the equation. There is therefore no rotation of the polarisation when the X-rays are scattered by the magnetic ion unlike the previous case in section 1.1.1. The equation describing the intensity is now

$$\begin{aligned} I = \pi^2_{CR22} + \pi^2_{CI22} + \pi^2_{MR22} + \pi^2_{MI22} \\ +2\pi_{CR22}\pi_{MR22} - 2\pi_{CI22}\pi_{MI22} \end{aligned} \tag{24}$$

thus demonstrating that the dependence of the scattered intensity is still quadratic but with a linear component. The relative size of the linear and quadratic dependence will depend on the sizes of the imaginary component and real component of the charge scattering which in turn will depend on the energy of the incident beam.

1.2. Circular Polarisation

1.2.1. Case 3: Moment in the Scattering Plane In the case of circularly polarised X-rays the amplitudes can be modelled as two orthogonal polarisations phase shifted by $\pi/2$ radians. Using the expressions in Equation 2 the two helicities for the incident beam can be represented as

$$
\begin{aligned}
P_i^{+} &= \sigma_i + i\pi_i \\
P_i^{-} &= \sigma_i - i\pi_i
\end{aligned} \tag{25}
$$

Here the results from the two helicities are distinguished using the indices + and −. It is now important that we include the phase difference i from the incident beam with a component in the scattering plane (π). The generalised equation (from Equation 6) for circular polarisation, including the $\pi/2$ radians phase change, will take the form

$$
\begin{aligned}
f^{+} &= \begin{pmatrix} \sigma_i \to \sigma_f & 0 \\ 0 & i\pi_i \to i\pi_f \end{pmatrix} F^{(0)} - i \begin{pmatrix} \sigma_i \to 0 & i\pi_i \to i\sigma_f \\ \sigma_i \to \pi_f & i\pi_i \to i\pi_f \end{pmatrix} \cdot MF^{(1)} \\
f^{-} &= \begin{pmatrix} \sigma_i \to \sigma_f & 0 \\ 0 & -i\pi_i \to -i\pi_f \end{pmatrix} F^{(0)} - i \begin{pmatrix} \sigma_i \to 0 & -i\pi_i \to -i\sigma_f \\ \sigma_i \to \pi_f & -i\pi_i \to -i\pi_f \end{pmatrix} \cdot MF^{(1)}
\end{aligned} \tag{26}
$$

for the two opposite helicities respectively. The different helicities result in different signs for the right hand terms in each matrix; these terms originate from the π incident terms.

To work out the helicities for the outgoing beam we use Equation 20 with the definitions given by Equation(9-14) but include the phase differences imposed by the circularly polarised beam shown in the representation of Equation 26. They are written out as follows:

$$
\begin{aligned}
f^{+} &= \begin{pmatrix} \sigma_{CR11} & 0 \\ 0 & i\pi_{CR22} \end{pmatrix} + i \begin{pmatrix} \sigma_{CI11} & 0 \\ 0 & i\pi_{CI22} \end{pmatrix} + \begin{pmatrix} 0 & i\sigma_{MR12} \\ \pi_{MR21} & i\pi_{MR22} \end{pmatrix} - i \begin{pmatrix} 0 & i\sigma_{MI12} \\ \pi_{MI21} & i\pi_{MI22} \end{pmatrix} \\
f^{-} &= \begin{pmatrix} \sigma_{CR11} & 0 \\ 0 & -i\pi_{CR22} \end{pmatrix} + i \begin{pmatrix} \sigma_{CI11} & 0 \\ 0 & -i\pi_{CI22} \end{pmatrix} + \begin{pmatrix} 0 & -i\sigma_{MR12} \\ \pi_{MR21} & -i\pi_{MR22} \end{pmatrix} - i \begin{pmatrix} 0 & -i\sigma_{MI12} \\ \pi_{MI21} & -i\pi_{MI22} \end{pmatrix}.
\end{aligned} \tag{27}
$$

The phase difference of $\pi/2$ radians before the magnetic part of the form factor now becomes important.

$$
\begin{aligned}
I^{+} = {} & \sigma_{CR11}^2 + \pi_{CR22}^2 + \sigma_{CI11}^2 + \pi_{CI22}^2 + \sigma_{MR12}^2 + \pi_{MR21}^2 + \sigma_{MI12}^2 + \pi_{MI21}^2 + \pi_{MR22}^2 + \pi_{MI22}^2 \\
& +2(\sigma_{CR11}\sigma_{MI12} - \pi_{CR22}\pi_{MI21} + \sigma_{CI11}\sigma_{MR12} - \pi_{CI22}\pi_{MR21}) \\
& -2(\pi_{MR22}\pi_{MI21} - \pi_{MI22}\pi_{MR21}) \\
& +2(\pi_{CR22}\pi_{MR22} - \pi_{CI22}\pi_{MI22}) \\
I^{-} = {} & \sigma_{CR11}^2 + \pi_{CR22}^2 + \sigma_{CI11}^2 + \pi_{CI22}^2 + \sigma_{MR12}^2 + \pi_{MR21}^2 + \sigma_{MI12}^2 + \pi_{MI21}^2 + \pi_{MR22}^2 + \pi_{MI22}^2 \\
& -2(\sigma_{CR11}\sigma_{MI12} - \pi_{CR22}\pi_{MI21} + \sigma_{CI11}\sigma_{MR12} - \pi_{CI22}\pi_{MR21}) \\
& +2(\pi_{MR22}\pi_{MI21} - \pi_{MI22}\pi_{MR21}) \\
& +2(\pi_{CR22}\pi_{MR22} - \pi_{CI22}\pi_{MI22})
\end{aligned}
\tag{28}
$$

This is the general case for circular polarisation. This can be simplified by keeping the moments in the scattering plane since the terms containing π_{MR22} and π_{MI22} are zero (these terms are only finite with a moment out of the scattering plane); Equation 28 then simplifies to the following:

$$
\begin{aligned}
I^{+} = {} & \sigma_{CR11}^2 + \pi_{CR22}^2 + \sigma_{CI11}^2 + \pi_{CI22}^2 + \sigma_{MR12}^2 + \pi_{MR21}^2 + \sigma_{MI12}^2 + \pi_{MI21}^2 \\
& +2(\sigma_{CR11}\sigma_{MI12} - \pi_{CR22}\pi_{MI21} + \sigma_{CI11}\sigma_{MR12} - \pi_{CI22}\pi_{MR21}) \\
I^{-} = {} & \sigma_{CR11}^2 + \pi_{CR22}^2 + \sigma_{CI11}^2 + \pi_{CI22}^2 + \sigma_{MR12}^2 + \pi_{MR21}^2 + \sigma_{MI12}^2 + \pi_{MI21}^2 \\
& -2(\sigma_{CR11}\sigma_{MI12} - \pi_{CR22}\pi_{MI21} + \sigma_{CI11}\sigma_{MR12} - \pi_{CI22}\pi_{MR21})
\end{aligned}
\tag{29}
$$

The first four terms σ_{CR11}, π_{CR22}, σ_{CI11} and π_{CI22} are charge terms and do not change with magnetic field. The next four terms σ_{MR12}, π_{MR21}, σ_{MI12} and π_{MI21} are quadratic in magnetic moment and are independent of the helicity of the beam. The last four terms $\sigma_{CR11}\sigma_{MI12}$, $\pi_{CR22}\pi_{MI21}$, $\sigma_{CI11}\sigma_{MR12}$ and $\pi_{CI22}\pi_{MR21}$ are linear in magnetic moment. They are the result of interference between charge and magnetic

scattering caused by a combination of circular polarisation and the 90° phase difference between the charge and magnetic form factors. The sign of these linear terms is dependent on the helicity.

In order to remove the quadratic components it is prudent to measure the hysteresis curves at two helicities and then take the difference. This is shown in Fig. 3. The quadratic parts have been made more significant by making $F^{(1)} = 2F^{(0)}$ to highlight the potential problems with non-linearity.

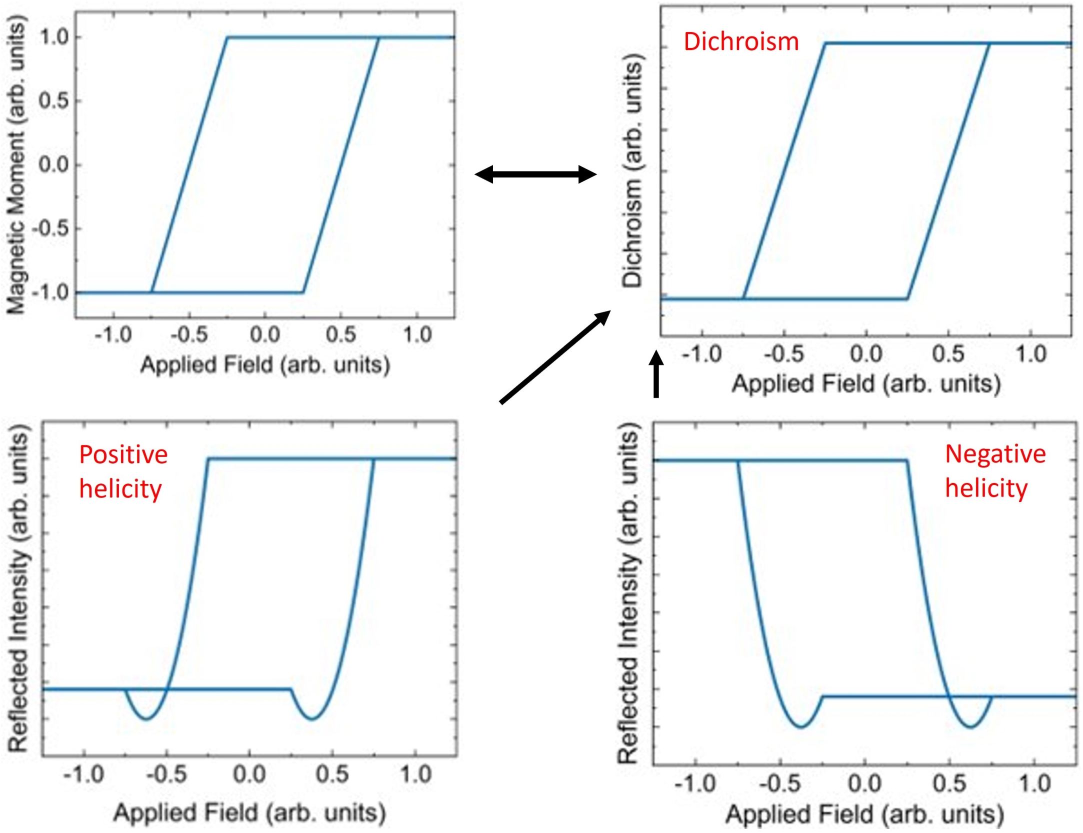

Fig. 3. At the top left is shown the hysteresis loop which will result in the reflectivity changes as shown in both plots at the bottom. These plots are the results of calculations done at the resonance (707eV) done with circular polarisation at opposite helicities (at the bottom). The calculations have been done with $F^{(1)} = 2F^{(0)}$ to add an increased quadratic component. Unlike the linear part this quadratic dependence does not change sign with helicity this means that the reflectivity hysteresis loops can be subtracted to remove this non-linear dependence which will give us the exact form of the hysteresis loop shown on the top right.

To switch the sense of the loops, without switching helicity and without going above a θ of 90° a phase is introduced. In scattering the exact phase is lost but since there are terms linearly dependent on magnetic moment these terms could be reversed by the effect of a phase. This can be done by introducing the phase in the Equations 28 and 29 respectively. The resulting modifications with the phase, designated as ϕ, are shown as Equations 30 and 31. The effect of the phase has been introduced as a

cosine which is only affecting the terms which are linear in magnetic moment. This is justified in our qualitative model since the sign change resulting from this phase would be lost in the quadratic terms.

$$\begin{aligned} I = \sigma_{CR11}^2 + \pi_{CR22}^2 + \sigma_{CI11}^2 + \pi_{CI22}^2 + \sigma_{MR12}^2 + \pi_{MR21}^2 + \sigma_{MI12}^2 + \pi_{MI21}^2 + \pi_{MR22}^2 + \pi_{MI22}^2 \\ +2(\sigma_{CR11}\sigma_{MI12} - \pi_{CR22}\pi_{MI21} + \sigma_{CI11}\sigma_{MR12} - \pi_{CI22}\pi_{MR21})\cos\phi \\ -2(\pi_{MR22}\pi_{MI21} - \pi_{MI22}\pi_{MR21}) \\ +2(\pi_{CR22}\pi_{MR22} - \pi_{CI22}\pi_{MI22})\cos\phi \end{aligned} \tag{30}$$

$$\begin{aligned} I = \sigma_{CR11}^2 + \pi_{CR22}^2 + \sigma_{CI11}^2 + \pi_{CI22}^2 + \sigma_{MR12}^2 + \pi_{MR21}^2 + \sigma_{MI12}^2 + \pi_{MI21}^2 \\ +2(\sigma_{CR11}\sigma_{MI12} - \pi_{CR22}\pi_{MI21} + \sigma_{CI11}\sigma_{MR12} - \pi_{CI22}\pi_{MR21})\cos\phi \end{aligned} \tag{31}$$

Equation 30 includes all the terms including those that would be present when there is a component of the magnetic moment out of the scattering plane π_{MR22} and π_{MI22}. This includes terms that are quadratic in magnetic moment i.e. $\pi_{MR22}\pi_{MI21}$ and $\pi_{MI22}\pi_{MR21}$. At low angles the π_{MR22} and π_{MI22} terms will be small due to their $\sin(2\theta)$ dependence. In this case equation 31 is a good approximation. It should be further noted though that the terms $\pm 2(\pi_{MR22}\pi_{MI21} - \pi_{MI22}\pi_{MR21})$ always equal zero. Furthermore if the magnetic reversal process is taking place in the scattering plane it can be assumed that, perpendicular to this plane, the net magnetic moment is zero. This means that terms linear in π_{MR22} and π_{MI22} are also zero. Therefore the last term in Equation 30 i.e. $2(\pi_{CR22}\pi_{MR22} - \pi_{CI22}\pi_{MI22})\cos\phi$ is ignored in this work.

1.2.2. Case 4: Moment Perpendicular to the Scattering Plane The Equation 28 can be simplified for having moments perpendicular to the scattering plane. Since the off-diagonal terms σ_{MR12}, π_{MR21}, σ_{MI12}, π_{MI21} only depend on the moments in the scattering plane these can all be set to zero. In fact the only magnetic terms are the diagonal π_{MR22} and π_{MI22}, which depend only on the moments perpendicular to the scattering plane. The Equation 28 greatly simplifies to Equation 32.

$$
\begin{aligned}
I^{+} &= \sigma_{CR11}^2 + \pi_{CR22}^2 + \sigma_{CI11}^2 + \pi_{CI22}^2 + \pi_{MR22}^2 + \pi_{MI22}^2 \\
&\quad +2(\pi_{CR22}\pi_{MR22} - \pi_{CI22}\pi_{MI22}) \\
I^{-} &= \sigma_{CR11}^2 + \pi_{CR22}^2 + \sigma_{CI11}^2 + \pi_{CI22}^2 + \pi_{MR22}^2 + \pi_{MI22}^2 \\
&\quad +2(\pi_{CR22}\pi_{MR22} - \pi_{CI22}\pi_{MI22})
\end{aligned}
\tag{32}
$$

The equations for both helicities are identical. Also noticeable is that Equation 32 is identical to that of Equation 24 apart from the additional charge terms σ_{CR11} and σ_{CI11} which do not change with applied field. This equation is also describing applied fields perpendicular to the scattering plane but for linear polarisation. Since it is only the π polarisation that is sensitive to this direction of the magnetic moment it is only this part of the circularly polarised wave that is contributing to the exchange of angular momentum between the incoming and outgoing beam. Since the σ polarisation is not sensitive to this direction the helicity of the beam is irrelevant for this case.

2. Modelling the Rotation of the Moments

Most of the models in the paper only change the moment in one dimension. That change is either parallel or perpendicular to the scattering plane. A more realistic model would involve rotating the moments at an angle to the scattering plane. This has been done to by having the moments rotate from 0 to 180° then back to 0°. To demonstrate this we have plotted the size of the moments projected both in the

scattering plane and perpendicular to the scattering plane during this process in Fig. 4. The moment parallel to the scattering plane will depend on the cosine of the angle to the scattering plane. As this angle varies from 0 to 180° during the switching (0 to 180° from 0.25 to 0.75 arbitrary units and then back from 180 to 0° from -0.25 to -0.75) the projection shown in the plot shows a smooth cosine function around the coercivities as shown on the left of Fig. 4. Plotted next to it is the projection of the moment perpendicular to the scattering plane. This varies sinusoidally with the angle to the scattering plane around the coercivities (-0.75 to -0.25 and 0.25 and 0.75) and is zero at all other points.

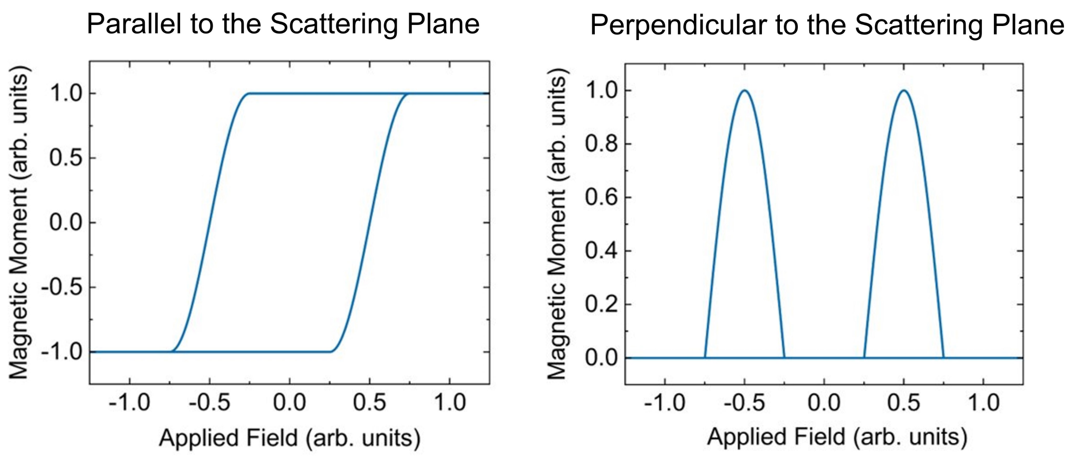

Fig. 4. The model used to simulate the magnetic moment rotating constantly from parallel to the scattering plane, through perpendicular then back to parallel (but in opposite direction) i.e varies between 0° and 180°. The projection of the moment parallel to the scattering plane is shown on the left. This will depend on the cosine of a constantly varying angle which varies around the coercivity between an apllied field of 0.25 and 0.75 arbitrary units and between -0.25 and 00.75 arbitrary units. On the right is shown the projection of the moment perpendicular to the scattering plane for the same process which depends on the sine of this angle around the coercivity.

References

Brück, S. (2009).
URL: *https://api.semanticscholar.org/CorpusID:93734541*

Hannon, J. P., Trammell, G. T., Blume, M. & Gibbs, D. (1988). *Physical Review Letters*, **61**(10), 1245.
URL: *https://journals.aps.org/prl/abstract/10.1103/PhysRevLett.61.1245*

HENKE, B., GULLIKSON, E. & DAVIS, J. (1993). *ATOMIC DATA AND NUCLEAR DATA TABLES*, **54**(2), 181–342.

Hill, J., McMorrow, D. & IUCr (1996). *urn:issn:0108-7673*, **52**(2), 236–244.
URL: *//scripts.iucr.org/cgi-bin/paper?sp0084*

iucr
